# Prevalence and pattern of transfusion transmitted infections among blood donors visiting a tertiary care hospital, Pakistan

**DOI:** 10.1186/s12879-026-13030-1

**Published:** 2026-03-09

**Authors:** Komal Iqbal, Sadia Farhad, Malik Shayan Ali Khan, Muhammad Hassan Jan, Inbasat Zahra Khan, Muhammad Umair, Bilal Hassan, Rafiullah Hotak

**Affiliations:** 1 Pathology Department, MTI-MMC, Mardan, Pakistan; 2 Hematology, MTI-MMC, Mardan, Pakistan; 3 Bacha Khan Medical College, Mardan, Pakistan; 4 Ayub Medical College, Abbottabad, Pakistan; 5 Bacha Khan College of Dentistry, MTI-MMC, Mardan, Pakistan; 6 Faculty of Medicine, Nangarhar University, Jalalabad, 2601 Nangarhar Province Afghanistan

**Keywords:** Transfusion-transmitted infections, Blood donors, Hepatitis B virus, Hepatitis C virus, Human immunodeficiency virus, Pakistan

## Abstract

**Objectives:**

This study aimed to assess the prevalence and patterns of HIV, HBV, HCV, and syphilis among blood donors in Pakistan.

**Methods:**

Data records of all registered blood donors (*n* = 63,847) during August 2020-April 2025, at a blood transfusion center in a tertiary care hospital were retrospectively analyzed. Prevalence of the seropositive donors for HIV, HCV, HBV and Syphilis was analyzed.

**Results:**

The donors ranged in age from 18 years to over 65 years, with the vast majority being male (99.47%). Overall prevalence of TTIs was 2.04% in the donor population. Hepatitis B virus (HBV) was the most prevalent TTI (1.12%; 95% CI: 1.04–1.20), followed by hepatitis C virus (HCV) (0.49%; 95% CI: 0.44–0.55), syphilis (0.36%; 95% CI: 0.32–0.41), and human immunodeficiency virus (HIV) (0.07%; 95% CI: 0.05–0.09) respectively. Replacement donations accounted for 59.34% of the total. The largest proportion of donors (48.7%) were aged 26–35 years. The highest annual donation volume occurred in 2023, with 13,534 donors. The most frequent coinfections were HBV–HCV and HBV–syphilis, with seven cases each.

**Conclusion:**

Among blood donors, HBV was the most prevalent transfusion-transmitted infection, followed by HCV.

**Clinical trial number:**

Not applicable.

## Introduction

Blood transfusion is a critical, life-saving intervention widely employed in clinical settings to manage various conditions, including anemia, traumatic injuries, major surgeries, malignancies, and hematologic disorders. Voluntary blood donation forms the foundation of modern healthcare and represents a generous act that can save numerous lives [1]. Nevertheless, ensuring the availability of safe blood and blood products continues to be a significant global challenge, especially in developing nations [2–5]. Despite advancements, unsafe transfusion practices can lead to avoidable complications, compromising patient safety. The 2020 SHOT (Serious Hazards of Transfusion) report indicates that human error remains the leading cause of transfusion-related incidents [6]. Blood transfusion also carries the risk of transmitting serious infections, including hepatitis B virus (HBV), hepatitis C virus (HCV), human immunodeficiency virus (HIV), syphilis, cytomegalovirus (CMV), herpes simplex virus (HSV), Epstein–Barr virus (EBV), and parasitic infections such as Toxoplasma gondii and malaria [7].

To minimize such risks, the World Health Organization (WHO) recommends mandatory serological screening of all donated blood for major transfusion-transmissible infections [3]. Infections such as HBV, HCV, and HIV pose significant public health concerns due to their long asymptomatic phases, during which infected individuals may unknowingly donate blood [8].

Accordingly, WHO strongly emphasizes routine testing of all blood donations—a practice now mandated and regularly implemented in most blood banks [9]. Still, transfusion-transmitted infections (TTIs) continue to pose a substantial threat, particularly in low- and middle-income countries where healthcare resources and testing capabilities remain limited [10]. Globally, the prevalence of transfusion-transmitted infections (TTIs) in donated blood varies considerably, with HIV ranging from 0.003% to 1.08%, HBV from 0.03% to 3.70%, HCV from 0.02% to 1.03%, and syphilis from 0.05% to 0.90% [9]. Among these, hepatitis B virus (HBV) remains the most common TTI in donor populations, with a steadily increasing prevalence over recent years [11]. Treponema pallidum, the bacterium responsible for syphilis, is considered the most medically significant bacterial agent causing TTIs [12].

The risk of transmission of blood-borne infections is notably higher in developing nations, primarily due to the higher prevalence of infectious agents among donors and suboptimal blood screening practices [13]. The primary pathogens responsible for transfusion-transmitted infections (TTIs) include human immunodeficiency virus (HIV), hepatitis B virus (HBV), hepatitis C virus (HCV), *Treponema pallidum* (syphilis), and the parasite causing malaria [14].

Studies indicate that the majority of blood donors are male (approximately 80.1%), with a median age of 23 years (IQR = 8 years) [15]. TTIs are most commonly detected in young adult donors [16]. Moreover, HBV infection shows a significant association with male donors aged 25 to 44 years, while HIV infection is more prevalent among female donors aged 35 years and above. Family or replacement donors also carry a notably higher burden of all four major TTIs [17].

Individuals aged 40–49 years are approximately twice as likely to contract HBV compared to those under 20 years of age (OR = 2.2; 95% CI: 1.17–4.04; *p* = 0.013) [17]. Recent advances in viral vaccine development highlight innovative approaches aimed at preventing major transfusion-transmissible infections such as HBV, HCV, HIV, and syphilis [18]. Recent studies emphasize that combining vaccine development with robust donor screening programs is essential to sustainably reduce transfusion-related infection risks, particularly in high-burden regions [19].

In Pakistan, a recent study reported a high overall TTI prevalence of 4.61% (1,929 cases out of 41,817 donations), with replacement donors showing a higher rate of 5.42% (1,057 out of 19,474 donations) and a notably low participation rate among female donors [20]. Currently, only 10%–13% of blood donations in Pakistan are voluntary [21]. This highlights the urgent need for future research to comprehensively assess the prevalence and patterns of TTIs in order to better understand the current trends and improve preventive strategies [22].

Despite improvements in screening technologies and the implementation of WHO-recommended protocols for infectious disease testing in blood banks, transfusion-transmitted infections (TTIs) remain a significant threat to transfusion safety [21]. This ongoing risk is largely attributed to systemic limitations, infrastructural constraints, and low public awareness [22]. Recent statistics from Pakistan reveal a concerning TTI prevalence among blood donors—4.61% across 41,817 donations, with replacement donors exhibiting an even higher rate of 5.42% [20]. These figures highlight deficiencies in voluntary blood donation initiatives and inadequacies in current donor screening practices. With only 10% to 13% of donations coming from voluntary, non-remunerated donors, the overreliance on family or paid donors—who are often associated with higher infection risks—further exacerbates the problem [21].

While similar challenges are documented in other developing nations, region-specific epidemiological data are essential to shape effective local health policies and guide resource allocation. The World Health Organization strongly encourages countries to collect and utilize localized data to support the development of blood safety initiatives. However, in Pakistan, particularly in smaller cities such as Mardan, there is a notable lack of updated data on TTIs, especially when stratified by age, gender, and donor type.

Weak surveillance systems in these regions hinder evidence-based policy-making and intervention planning. This study seeks to address that gap by providing current, region-specific data on the prevalence and demographic distribution of TTIs among blood donors at MMC-MTI Mardan. The findings aim to contribute to safer transfusion practices, promote voluntary donation, and inform improved screening protocols. Ultimately, this research could lay the groundwork for future national surveillance efforts and policy reforms aimed at reducing transfusion-associated risks within Pakistan’s healthcare system.

## Methods

The data used in this research pertain to the period from August 2020 to April 2025. The electronic records of the blood donors registered at the blood bank of a tertiary care hospital were assessed. The study protocol was reviewed and approved by the Ethical Committee of Bacha Khan Medical College, Mardan (Ref No. 809/BKMC), under the authority of the Advance Studies and Research Board (ASRB), the primary governing body for research at BKMC, MTI Mardan. The Ethics Committee of Bacha Khan Medical College, MTI-MMC Mardan formally waived the requirement for informed consent because the study involved retrospective analysis of fully de-identified donor records and posed minimal risk to participants, in accordance with institutional and national ethical guidelines. This approval permitted access to donor screening data retrospectively, from April 2025 back to August 2020. The hospital’s Health Management Information System (HMIS) contained data on blood donors from August 2020 onwards. All blood donation samples were screened for four transfusion-transmissible infections (TTIs): HIV, HCV, HBV, and Syphilis. All blood donors (voluntary and replacement) from 2020 to 2025, who had body weight of ≥ 50 kg were included. Anemic donors (hemoglobin < 12.5 g/dl for females and < 13.5 g/dl for males) were excluded and anemia status was confirmed as part of the routine pre-donation Complete Blood Count (CBC) using the Abbott Cell-Dyn Ruby 7090 G4 hematology analyzer (7-part differential). Donors with incomplete records were excluded from this analysis.

As per the institutions` practice, all blood donors underwent pre-donation counseling conducted by a staff nurse. Before donation, each individual completed a form with basic sociodemographic details and type of donation (voluntary or replacement). For serological screening, we used CLIA on the Abbott Architect i1000 with a cut-off index (COI) of < 1.0 for all markers to define non-reactivity. All initially reactive samples were retested in duplicate, and those that remained reactive upon repeat testing were subsequently confirmed by ELISA, following standard protocols.

Routine donor screening in this study relied on HBsAg due to cost and feasibility constraints [3, 7]. However, HBV DNA is the most reliable indicator of infectivity; Yin et al. showed that high maternal HBV DNA levels markedly increase intrauterine transmission risk, reinforcing its strong correlation with transmissibility, including in transfusion settings [23].

The collected data was analyzed using SPSS version 27. P value of less than 0.05 was considered significant. Descriptive and analytical statistics were applied for qualitative variables like gender, type of donation, age group and infection type etc. For each variable, frequency and percentages have been calculated.

## Results

Between August 2020 and April 2025, a total of 63,847 blood donors were screened for four major transfusion-transmitted infections (TTIs)—hepatitis B virus (HBV), human immunodeficiency virus (HIV), hepatitis C virus (HCV), and syphilis—at the tertiary care hospital.

**Table 1:**
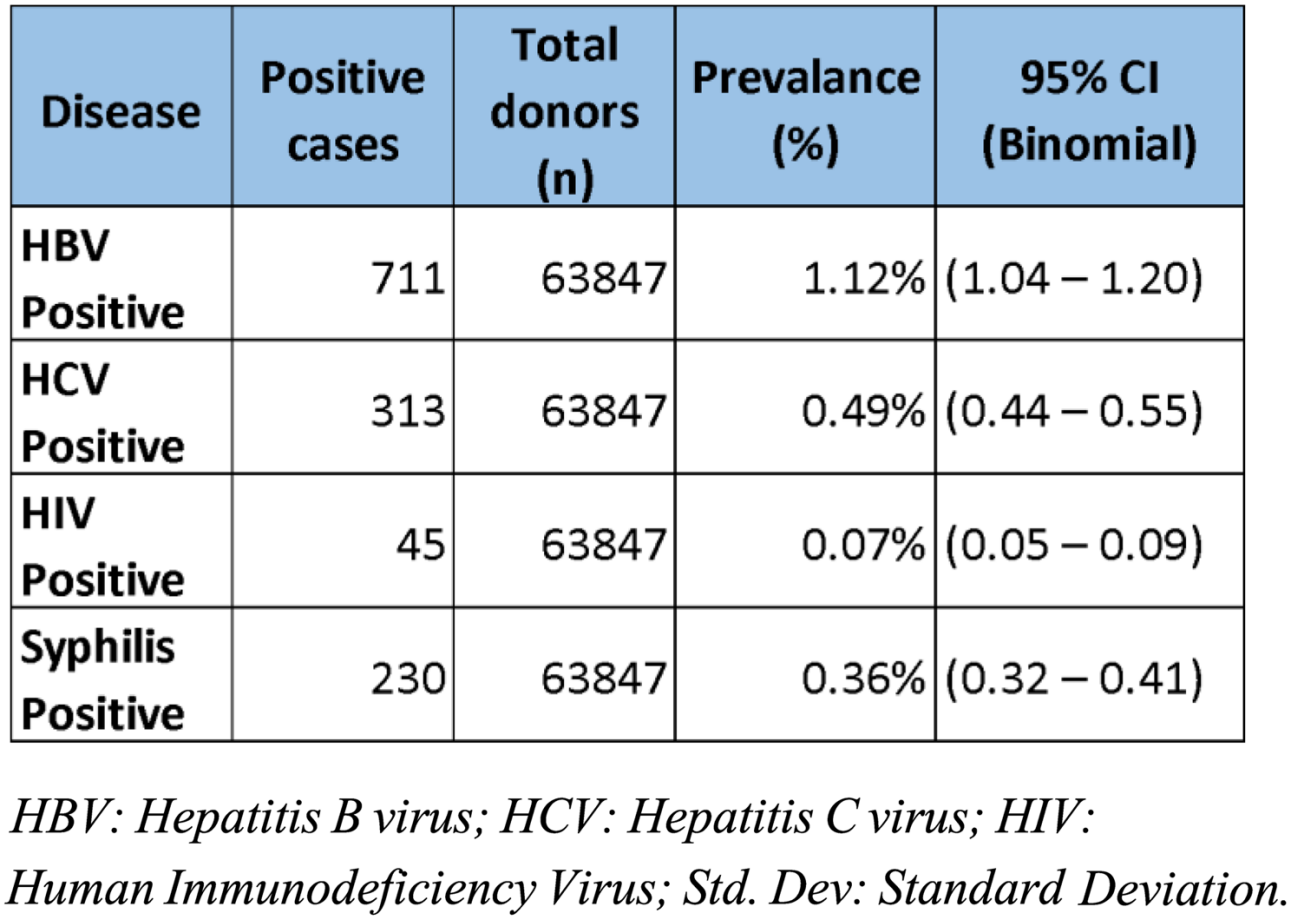
Descriptive Statistics of Transfusion-Transmissible Infections (HBV, HCV, HIV, and Syphilis) Among Blood Donors, Showing number of positive cases, total donors screened, calculated prevalence (%), 95% Confidence Intervals estimated using the exact binomial (Clopper-Pearson) method for each infection

Among the total 63,847 blood donors included in the study, the overall prevalence of transfusion-transmissible infections (TTIs) was 2.04%. The distribution of individual infections is presented in Table 1. Hepatitis B virus (HBV) was the most frequently detected infection, with 711 positive cases, corresponding to a prevalence of 1.12% (95% CI: 1.04–1.20). Hepatitis C virus (HCV) infection was identified in 313 donors (0.49%, 95% CI: 0.44–0.55), while human immunodeficiency virus (HIV) was found in 45 donors (0.07%, 95% CI: 0.05–0.09). Syphilis was positive in 230 donors (0.36%, 95% CI: 0.32–0.41). These findings indicate that HBV remains the predominant transfusion-transmissible infection among blood donors, followed by HCV, syphilis, and HIV, respectively.

**Table 2:**
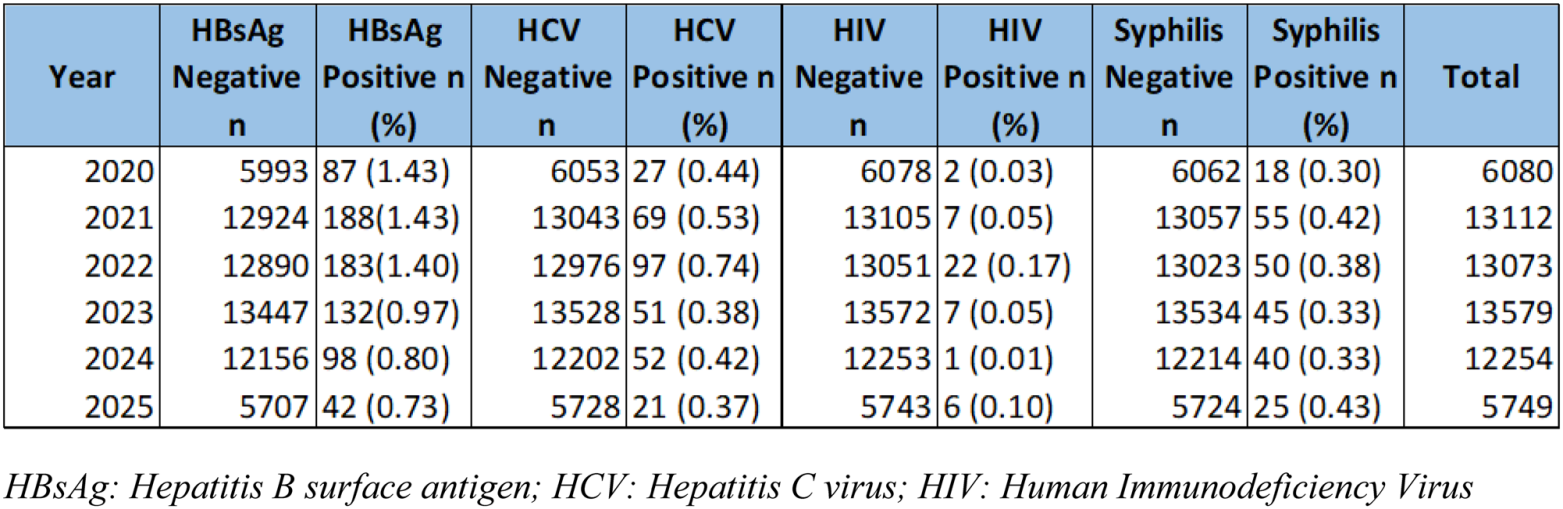
Year-wise seropositive cases of HBV, HCV, HIV and Syphilis infections in blood donors along with seronegative cases (*N* = 63,847). The total number of donations have been shown across every year

Table 2 presents the yearly distribution of transfusion-transmitted infections (TTIs) among blood donors from 2020 to 2025. The table summarizes the number and percentage of donors who tested seropositive for hepatitis B surface antigen (HBsAg), hepatitis C virus (HCV), human immunodeficiency virus (HIV), and syphilis. Over the six-year period, HBsAg sero positivity showed a gradual decline from 1.43% in 2020 to 0.73% in 2025. Similarly, the prevalence of HCV seropositivity decreased from 0.44% to 0.37%, while HIV seropositivity remained consistently low, ranging between 0.01% and 0.17%. Syphilis seropositivity fluctuated slightly, with rates between 0.30% and 0.43%. The total number of blood donors varied annually, reaching a peak of 13,575 in 2023 and decreasing to 5,749 in 2025. Overall, the data indicate a declining trend in most TTIs among blood donors during the study period.

Chi-square analysis showed a significant association between year and seropositivity for HBV (χ² = 46.82, df = 5, *p* < 0.001), HCV (χ² = 23.83, df = 5, *p* < 0.001), and HIV (χ² = 27.88, df = 5, *p* < 0.001), indicating fluctuating prevalence over time. In contrast, syphilis seropositivity did not vary significantly by year (χ² = 3.67, df = 5, *p* = 0.598). When considering all infections combined, the overall positivity also varied significantly across years (χ² = 61.80, df = 5, *p* < 0.001).

Binary logistic regression further confirmed the effect of year on TTI positivity. Using 2023 as the reference, donors in 2020 (OR = 0.74, *p* = 0.026), 2021 (OR = 0.67, *p* = 0.001), and 2022 (OR = 0.61, *p* < 0.001) had significantly lower odds of testing seropositive, while no significant differences were observed for 2024 (OR = 0.95, *p* = 0.641) or 2025 (OR = 1.05, *p* = 0.703). The overall model was statistically significant (χ² = 60.78, df = 5, *p* < 0.001) but explained a very small proportion of variance (Nagelkerke R² = 0.004). Although the regression model reached statistical significance, the low Nagelkerke R² value (0.004) indicates limited explanatory power. This suggests that the year of donation alone explains only a small portion of the variation in TTI positivity, and other unmeasured factors may contribute to the observed trends.

**Fig. 1:**
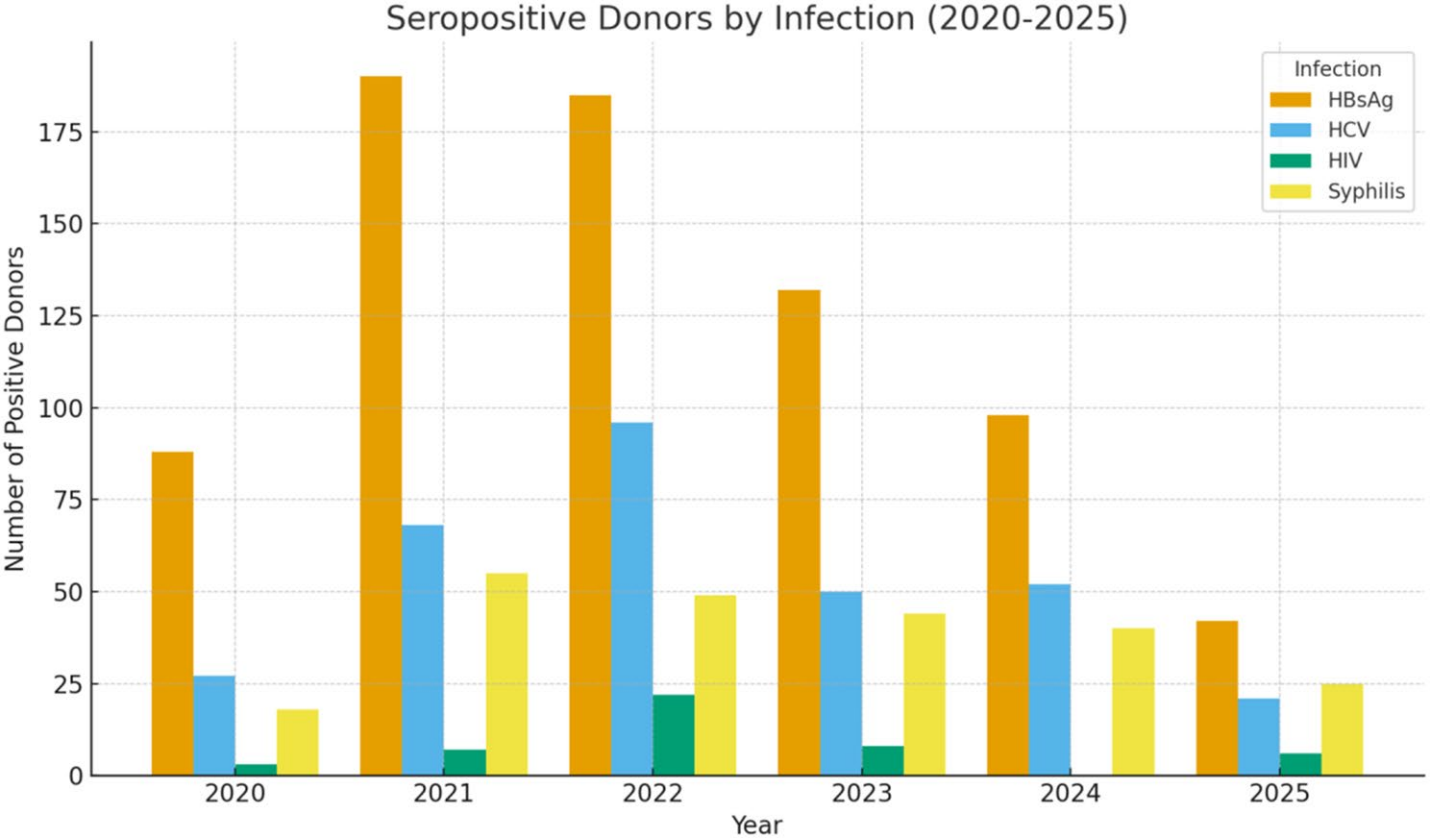
Bar chart showing the number of seropositive cases of HBsAg, HCV, HIV, and Syphilis among blood donors across five (5) years

Figure 1 illustrates yearly fluctuations in TTIs. HBV showed the highest burden overall, peaking in 2021 (≈ 188 cases) before gradually declining through 2025. HCV increased steadily from 2020, peaked in 2022 (≈ 97 cases), and then declined moderately in subsequent years. HIV remained the least prevalent infection, with a small rise in 2022 (≈ 22 cases) but mostly in single digits across other years. Syphilis showed a peak in 2021 (≈ 55 cases) followed by a gradual decrease to about 25 cases by 2025.

**Fig. 2:**
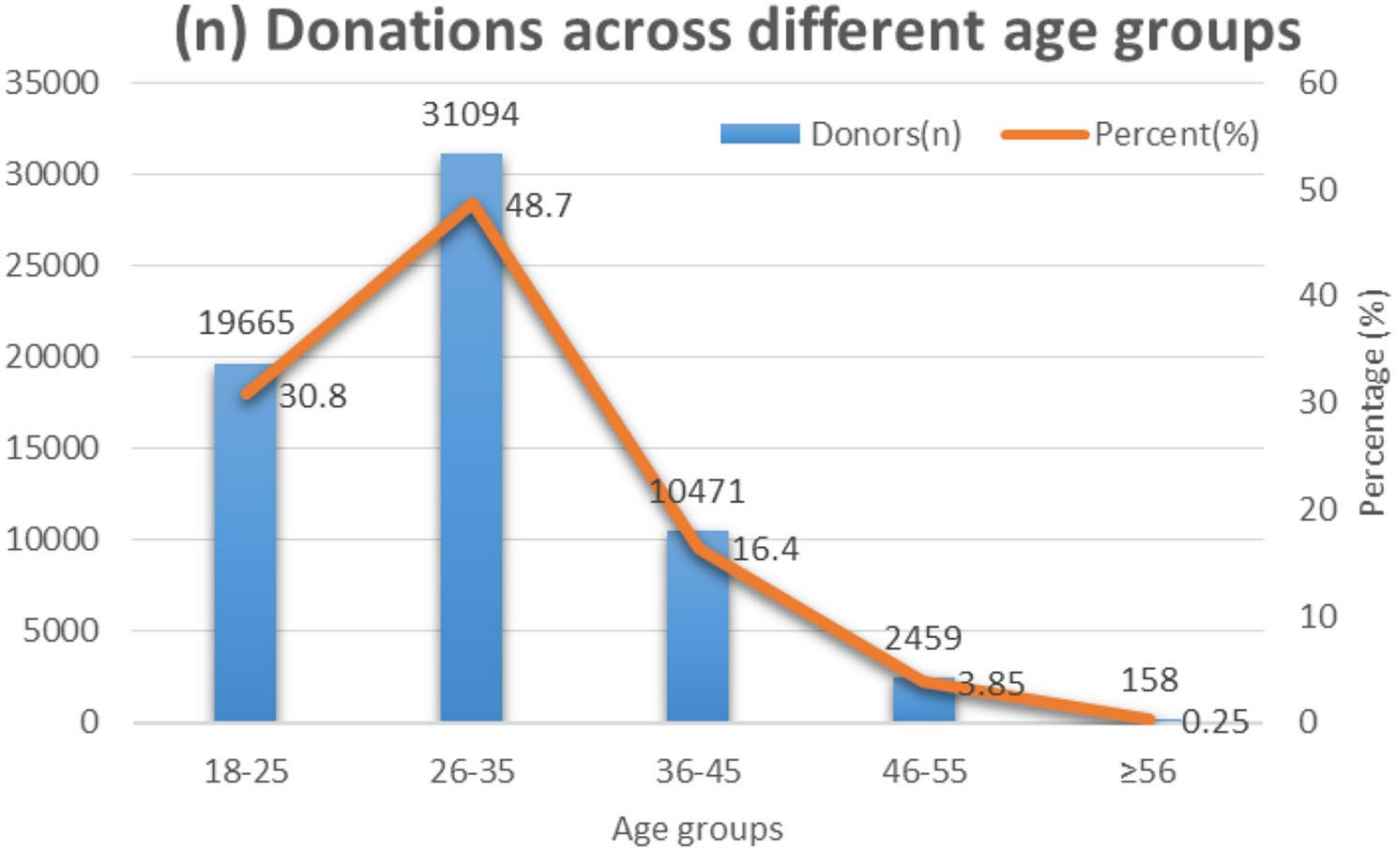
Total number of donations across different age groups along with percentages (%)

The number of donations were also analyzed across different age groups (Fig. 2). The 26–35 age group contributes the highest donor count and the greatest absolute numbers of all four infections. The 26–35 age group has the highest number and percentage of donors. The ≥ 56 age group has the lowest representation. The distribution is right-skewed (most donors are younger).

**Fig. 3:**
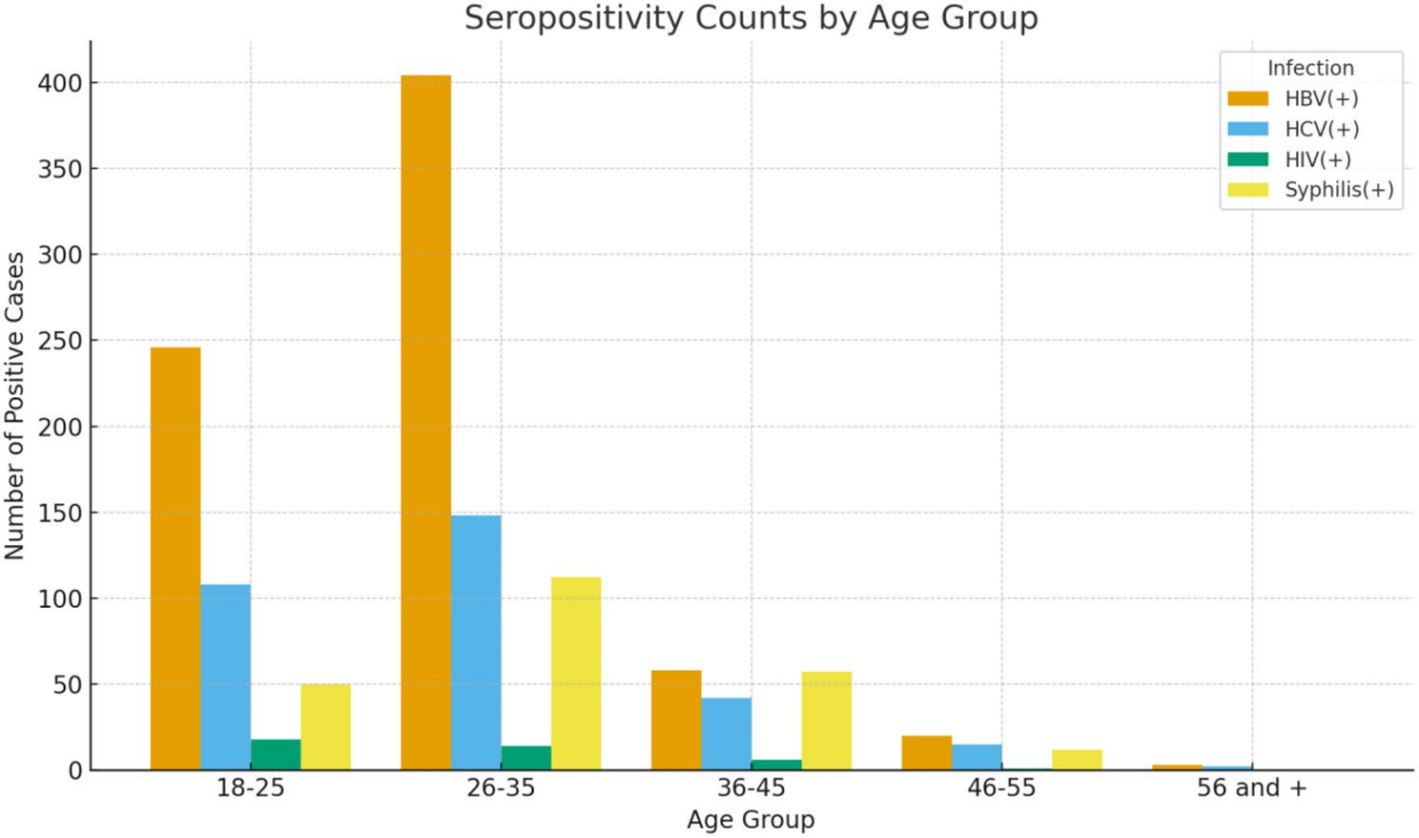
Frequency of seropositive cases across different age groups. (26–35 years) age group showed the highest burden of transfusion transmitted infections

Figure 3 shows that the 26–35 years age group carried the highest overall burden of transfusion-transmissible infections. HBV dominated seropositive findings across all age groups, with prevalence peaking in the 18–25 and 26–35 categories and then dropping sharply after 35 years. The association between age and HBV infection was statistically significant (*p* < 0.005). Syphilis also demonstrated a significant association with age (*p* < 0.005), being most common in the 36–45 group followed by 46–55 and 26–35 age groups respectively. Donor volume decreased substantially after 45 years, which paralleled the decline in seropositive cases. In contrast, HCV and HIV showed no significant association with age groups.

Chi-square analysis confirmed that TTI prevalence varied significantly across age groups (χ² = 68.744, *p* < 0.001). For regression analysis, the 46–55 and 56 + groups were combined into a single ≥ 46 years category due to the small number of donors in the oldest group. Multinomial logistic regression, using HBV as the reference infection and ≥ 46 years as the reference age category, was statistically significant (χ² = 61.62, df = 9, *p* < 0.001). Donors aged 26–35 had significantly lower odds of HCV seropositivity relative to HBV compared to those ≥ 46 years (OR = 0.499; 95% CI: 0.259–0.960; *p* = 0.037). For syphilis, donors aged 18–25 (OR = 0.360; 95% CI: 0.171–0.757; *p* = 0.007) and 26–35 (OR = 0.490; 95% CI: 0.241–0.999; *p* = 0.050) also showed lower odds relative to HBV than older donors. Age group did not significantly predict HIV seropositivity relative to HBV (all *p* > 0.05).

**Table 3:**
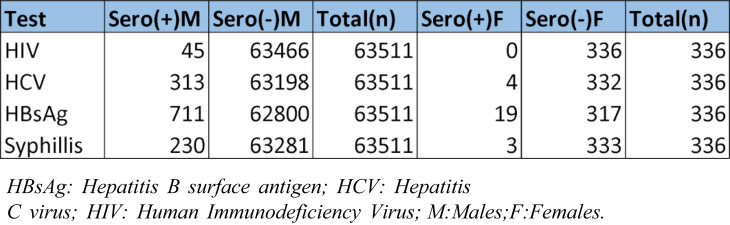
Gender-wise frequency of Transfusion transmitted infections with total number of Male and Female donors

The donor population was predominantly male (*n* = 63,511) compared to female donors (*n* = 336), totaling 63,847 donations. Hepatitis B surface antigen (HBsAg) was the most prevalent TTI in both genders, with a prevalence of 1.12% in males and 5.65% in females (Table 3). Among male donors, the most frequent infection was hepatitis B (1.12%), followed by HCV (0.49%), syphilis (0.36%), and HIV (0.07%). Among female donors, the most frequent infection was hepatitis B (5.65%), followed by HCV (1.19%) and syphilis (0.89%), while no HIV-seropositive cases were identified.

Statistical analysis revealed a significant association between overall TTI seropositivity and gender (*p* < 0.001). This significance likely reflects the large difference in sample sizes rather than a true biological difference in infection rates. However, when individual infections (HBV, HCV, HIV, and syphilis) were analyzed separately, no statistically significant associations with gender were observed (all *p* > 0.05). This suggests that gender influences the overall likelihood of TTI seropositivity but does not significantly affect the risk of specific infections, which may be explained by the limited number of female donors and small case counts within each subgroup.

**Table 4:**
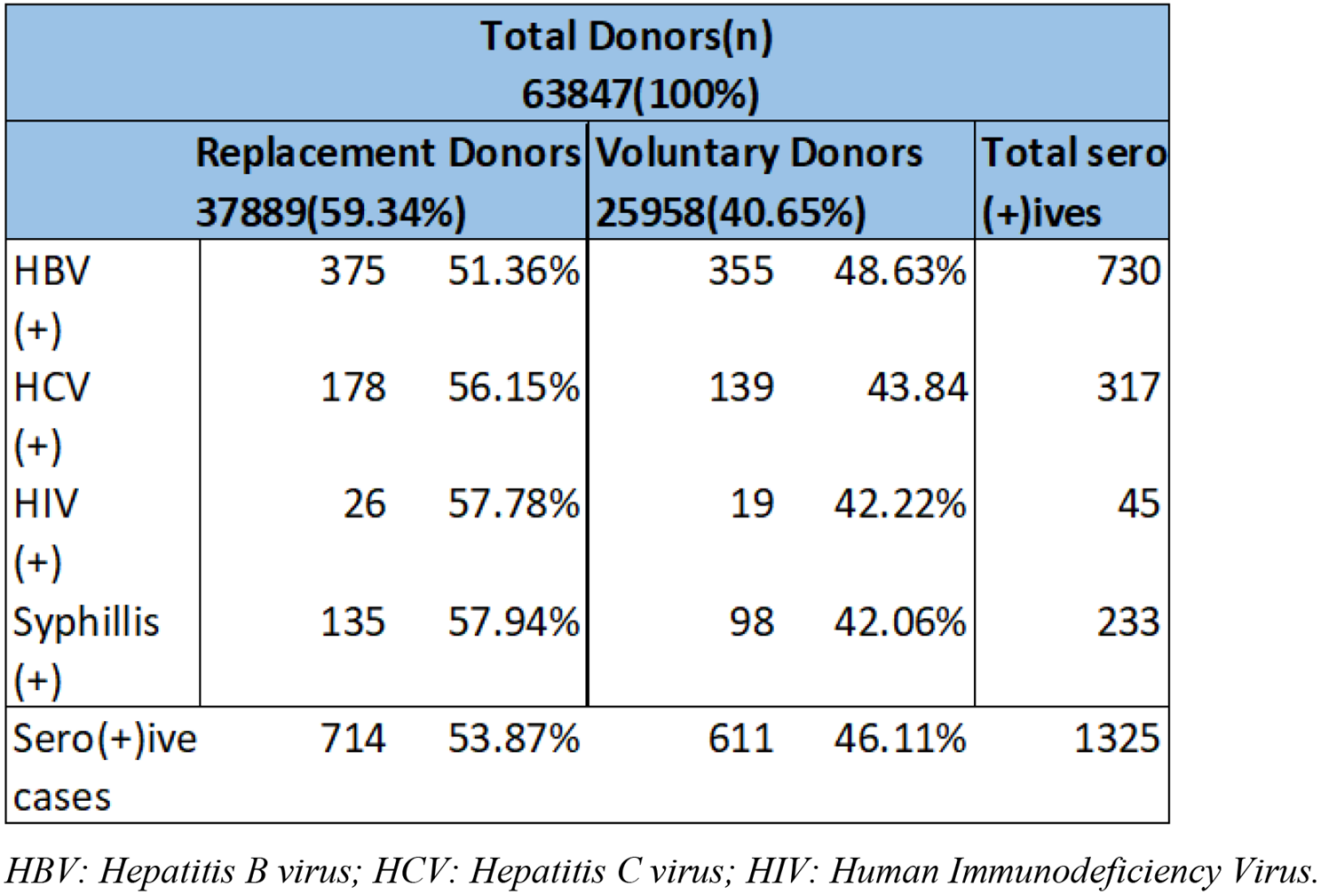
Comparison of seropositive cases among replacement and voluntary blood donors. Replacement donors showed a higher burden of TTIs, with statistically significant association for HBV and overall positivity

In Table 4, Analysis of the blood donors` data shows that the majority of the donations are Replacement in nature (59.34%) against Voluntary donations (40.65%). For each TTI, the number of seropositive cases are greater in Replacement donors than in Voluntary donors. Replacement donors contribute 714 seropositive cases versus 611 among voluntary donors. For every infection, the Replacement group edges higher, but the gap is smallest for HBV (375 vs. 355) and widest for HCV (178 vs. 139). HIV and syphilis show similar proportional splits, about 58% Replacement to 42% Voluntary.

**Fig. 4:**
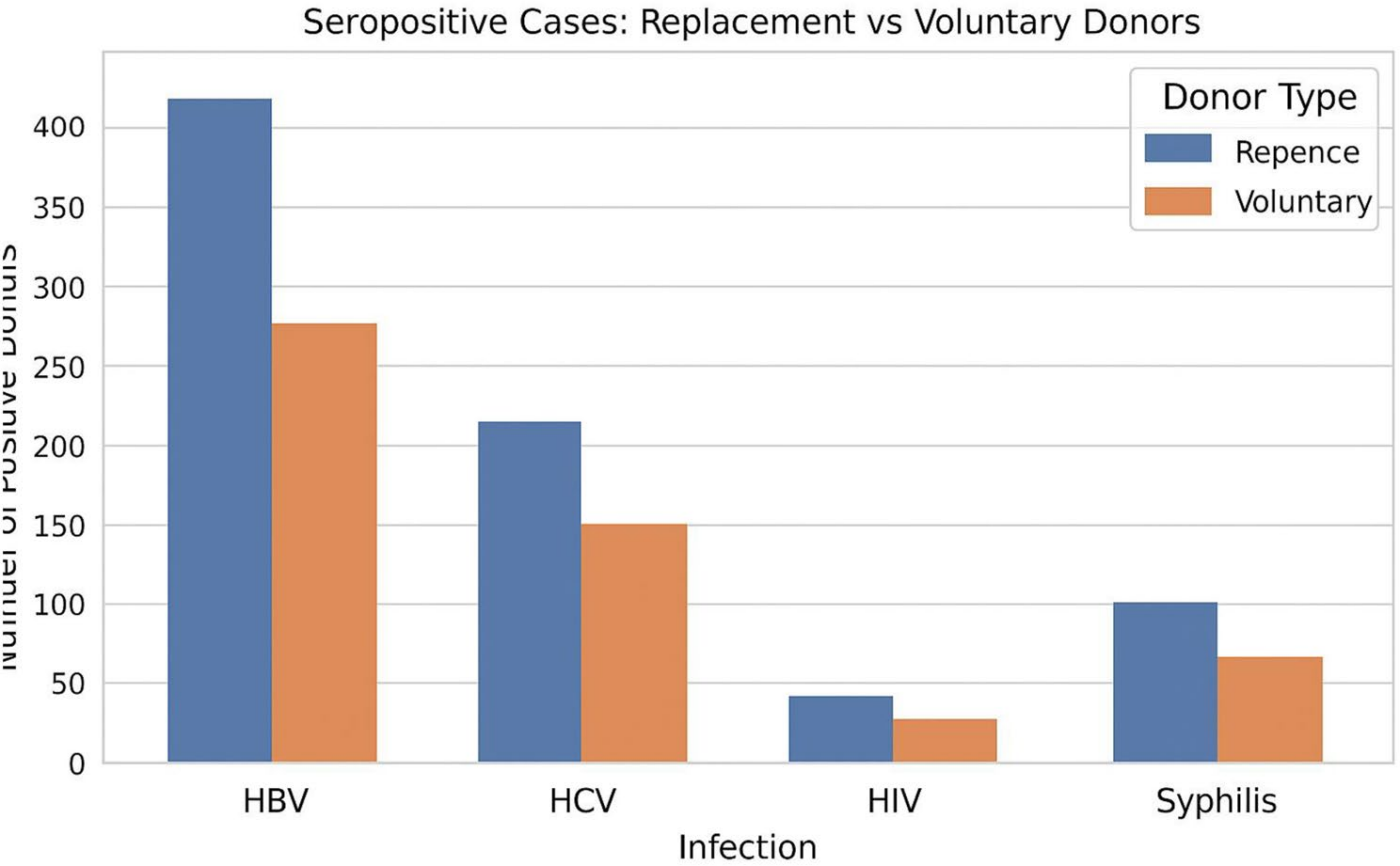
Trend of seropositive cases in replacement vs. voluntary donors

Overall, replacement donors carried a higher infectious burden despite comprising about 59% of the total donor pool. This supports the common observation that first-time or replacement donations pose greater screening risks than repeat voluntary donations. Inferential analysis showed significant associations for HBV (*p* = 0.000012) and overall seropositive cases (*p* = 0.000049), indicating that replacement donors were significantly more likely to be HBsAg-positive or seropositive in general. No significant differences by donor type were observed for HCV, HIV, or syphilis (Fig. 4).

Chi-square analysis confirmed a significant association between donation type and overall TTI seropositivity (χ² = 16.437, *p* < 0.001), with replacement donors showing higher seropositivity rates. Binary logistic regression further supported this, with a statistically significant model (− 2 Log likelihood = 16,569.505; *p* < 0.001), though it explained only a small proportion of variance (Cox & Snell R² = 0.001; Nagelkerke R² = 0.001). Donation type was a significant predictor (Wald = 16.37, *p* < 0.001), with voluntary donors having lower odds of TTI seropositivity compared to replacement donors (OR = 0.80; 95% CI: 0.72–0.89). These results reinforce that replacement donors represent a higher risk group for transfusion-transmissible infections. However, the very low R² values (Cox & Snell R² = 0.001; Nagelkerke R² = 0.001) indicate that the explanatory power of this model is minimal. While donor type was statistically significant, it accounts for only a small fraction of the variance in TTI seropositivity, implying that additional behavioral or demographic factors may play a larger role.

**Table 5:**
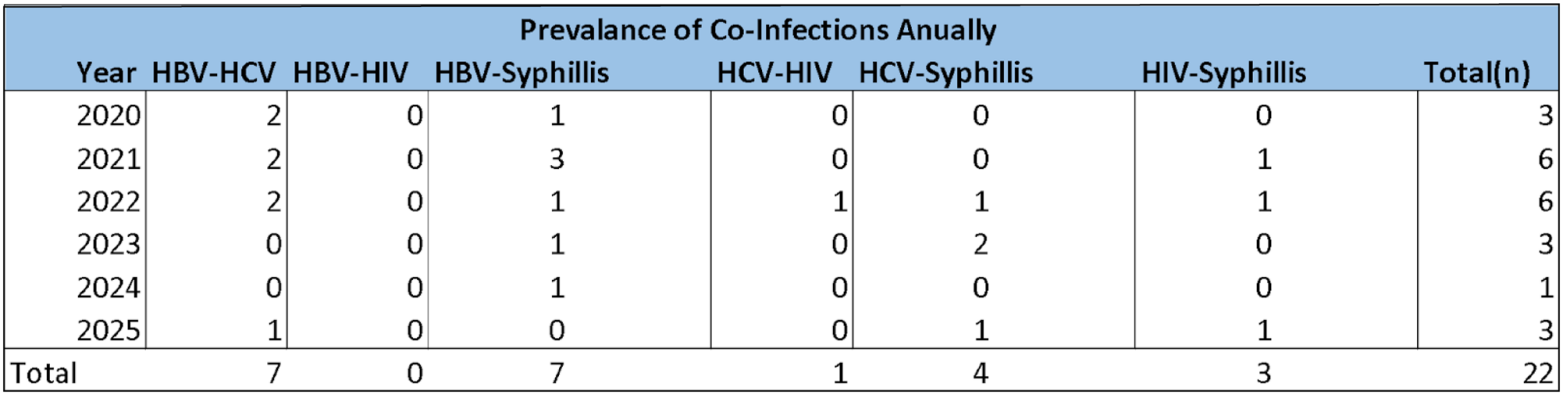
Annual pattern of different co-infections in blood donors

The annual prevalence and pattern of Co-infections has been analyzed (Table 5). The most prevalent Co-infections are HBV-HCV and HBV-Syphilis with overall (7) cases each. There has been no co-infection of HBV-HIV recorded.

## Discussion

This retrospective study aimed to assess the prevalence of transfusion-transmitted infections (TTIs) among blood donors in Pakistan. The study population included individuals who donated blood between 2020 and 2025 at the largest tertiary care hospital in the Mardan district. The hospital’s Blood Transfusion Services (BTS) collect over 13,000 units of blood annually, all of which are routinely screened for hepatitis C virus (HCV), hepatitis B virus (HBV), syphilis, and human immunodeficiency virus (HIV). The present analysis focuses on the distribution and frequency of TTIs among donors, with the goal of providing data to inform evidence-based strategies for enhancing blood safety at the national level.

We analyzed the data of 63,847 blood donors who were screened for four pathological entities (HIV, HCV, HBV and Syphilis) and overall prevalence of TTIs was 2.04% in the donor population. Other researches have shown prevalence of TTIs up to 5.44% which was much higher than the prevalence shown in our study [21–22]. A majority of the donors (31,094, 48.7%) belonged to the 26 to 35-year-old group followed by 19,665 (30.8%) donors who were ≤ 25 years, which is similar to a study conducted in Rawalpindi [24]. The ≥ 56 years age group had the lowest representation in our study.

The age distribution was right-skewed, with the majority of donors belonging to younger age groups, consistent with previous reports from Pakistan and other regional studies where blood donation is largely concentrated among young adults, with minimal participation from older individuals [24–26].HBV showed a significant association with age, with infections concentrated in the 18–35 age group, consistent with studies from Pakistan and Qatar that reported higher HBV prevalence among younger donors [24–27]. Syphilis, by contrast, was more common in middle-aged donors (36–55 years), a pattern also observed in Uganda and Gabon [15, 17]. These trends highlight the need for targeted interventions—HBV vaccination and awareness for younger adults, and syphilis screening reinforcement in middle-aged donor groups.

In our study, the predominance of male donors (99.47%) aligns with trends reported in other developing countries, where men consistently outnumber women in blood donation [25–27]. Female participation was exceptionally low (0.53%), which may be influenced by physiological and reproductive factors such as menstruation, pregnancy, and breastfeeding, consistent with prior studies highlighting these as common barriers to female donation [25, 26]. Hepatitis B surface antigen (HBsAg) remained the most frequent infection across both genders, corroborating regional and global findings that HBV continues to be the leading TTI among blood donors, despite vaccination efforts [3, 18, 19, 21].

The lack of statistically significant associations between gender and individual infections may be attributed to the small female sample size, limiting the power to detect differences within subgroups [9, 13, 14]. Our findings also emphasize the importance of targeted interventions to improve female donor participation and reinforce screening strategies for HBV and HCV, particularly in low-resource settings where blood safety remains a critical challenge [4, 5, 7, 8, 10]. Moreover, consistent with WHO guidelines, strengthening donor recruitment, vaccination coverage, and robust serological screening are vital to reducing TTI transmission and ensuring a safe and adequate blood supply [3, 12].

As far as year wise frequency of TTIS is concerned, HBV consistently showed the highest average prevalence (1.12%), followed by HCV (0.49%), syphilis (0.36%), and HIV (0.07%). This distribution aligns with findings from a regional study in Pakistan, where HBV was also reported as the most prevalent TTI and HIV the least [20]. Similarly, Oyedeji et al. [11] documented HBV as the leading infection across multiple years in Nigeria, reinforcing the global burden of hepatitis B in blood donor populations. In our data, syphilis showed a consistent presence and ranked second to HCV in most years, a trend that parallels the findings of Bhatti et al. [27] in Pakistan. These consistencies highlight the persistent dominance of HBV among TTIs, the relatively stable but concerning presence of syphilis, and the consistently low prevalence of HIV across different populations and settings.

HBV DNA is the most accurate marker of viral replication and infectivity [23], but its routine use in donor screening is limited by cost and feasibility [3]. Consequently, HBsAg remains the most practical marker for identifying current infection and transfusion risk, while anti-HBc IgM serves mainly to confirm acute infection or detect cases during the window period [28]. HBV infection itself is clinically diverse, ranging from acute and chronic hepatitis to fulminant or asymptomatic states; in the present study, only serological markers were assessed, as comprehensive staging requires molecular follow-up beyond our scope [29]. In addition, recent reports of severe acute hepatitis of unknown origin in children suggest that novel or immune-mediated mechanisms may also influence hepatitis epidemiology [30].

Immunization status critically influences the prevalence of transfusion-transmitted infections. Of the pathogens assessed in this study, only hepatitis B has an effective vaccine, incorporated into Pakistan’s Expanded Programme on Immunization in 2002 [21]. However, incomplete coverage has limited its protective impact [31, 32], which could be a contributing factor to the higher prevalence of hepatitis B observed in our study population. For HCV, HIV, and syphilis, no licensed vaccines are currently available, and prevention relies mainly on donor screening and safe practices [33].

The most prevalent Co-infections in our study were HBV-HCV and HBV-Syphilis with overall (7) cases each. In our study, no cases of HBV–HIV coinfection were observed. Likewise, the prevalence of HCV–HIV coinfection was nearly negligible (≈ 0.001%). These findings are in line with recent research, which consistently demonstrates that such co-infections are exceptionally rare among blood donor populations [27, 34].

Analysis of the blood donors` data showed that the majority of the donations were Replacement in nature (59.43%) against Voluntary donations (40.65%) comparable to a study conducted in the same province [20]. In our study, voluntary donors comprised **40.65%** of the total donations. This finding is comparable to a Peshawar study, which reported a voluntary donation rate of **53.4%** [35]. However, other regional studies from Karachi and Lahore reported rates below **20%**, where replacement donors remain predominant [36, 37]. For each TTI, the number of seropositive cases are greater in Replacement donors than in Voluntary donors similar to other studies [17–20]. HBsAg and total seropositive cases showed a statistically significant association with donor type (significant p values).

While our results indicate a measurable prevalence of transfusion-transmitted infections among blood donors in this single tertiary-care center, these findings do not imply direct causation nor do they represent the national prevalence. Larger, multicenter, and population-based studies are required to confirm and generalize these observations.

This study also has certain limitations. First, it was conducted at a single tertiary-care hospital in Khyber Pakhtunkhwa, Pakistan, which may not reflect the broader national population. Second, the donor population was predominantly male (99.47%), with only 0.53% female donors, limiting the ability to make meaningful conclusions regarding TTI prevalence among women in the region. Third, the retrospective design relied on routinely recorded blood bank data, which may be subject to reporting errors or incomplete records. Fourth, data on risk factors—such as socioeconomic status, education level, sexual practices, or vaccination history—were not available, restricting the ability to identify determinants of TTI prevalence. Lastly, the cross-sectional nature of the dataset limits causal inference and provides only a snapshot of trends during the study period.

## Conclusion

The Prevalence and Pattern of TTIs among blood donors in our study indicate that TTIs remain a significant risk to safer blood donation practices in Pakistan. Certain measures such as proper screening of the donated blood, filling of the Donors health questionnaire, discouragement of the replacement donations are important to enhance blood safety. The health policymakers should implement strategies to further improve safe blood donation practices in Pakistan. Furthermore, Hepatitis B contributed the major portion of TTIs in our donor population. This reflects the neglected need of Hepatitis B vaccination in the general population. The majority of blood donors were young individuals, particularly those under the age of 35. This underscores the importance of ensuring follow-up care for all seropositive donors—especially young men—by providing them with appropriate counseling and specialized medical treatment. Such interventions are crucial to prevent the further spread of potentially serious infections to their families and the broader community.

## Data Availability

The dataset used and analysed during current study are available from the corresponding author, Rafiullah Hotak, upon reasonable request.

## Abbreviations

TTI: Transfusion-Transmitted Infection
HBV: Hepatitis B Virus
HCV: Hepatitis C Virus
HIV: Human Immunodeficiency Virus
MMC: Mardan Medical Complex
MTI: Medical Teaching Institution
BKMC: Bacha Khan Medical College
WHO: World Health Organization
CLIA: Chemiluminescent Immunoassay
HBsAg: Hepatitis B Surface Antigen
HMIS: Health Management Information System
SPSS: Statistical Package for the Social Sciences
CI: Confidence Interval
